# Associations of iron status with apolipoproteins and lipid ratios: a cross-sectional study from the China Health and Nutrition Survey

**DOI:** 10.1186/s12944-020-01312-9

**Published:** 2020-06-16

**Authors:** Bowen Zhou, Huihui Ren, Xinrong Zhou, Gang Yuan

**Affiliations:** Department of Endocrinology and Metabolism, Tongji Hospital, Huazhong University of Science and Technology, Wuhan, 430030 Hubei China

**Keywords:** Iron, Ferritin, Transferrin, Soluble transferrin receptor, Apolipoprotein A1, Apolipoprotein B, Lipid ratio, Cardiometabolic risk

## Abstract

**Background:**

Iron overload has been found to be related with various cardiometabolic disorders, like dyslipidemia, metabolic syndrome, and diabetes. The disturbance of the iron status and lipid metabolism can contribute to organ damage such as atherosclerotic plaque growth and instability. An assessment on the associations of iron status with apolipoproteins and lipid ratios would be informative for maintenance of metabolic homeostasis and hinderance of disease progression. Hence, this study aims to establish the relationships of iron status with apolipoproteins and lipid ratios.

**Methods:**

A cross-sectional study of 7540 adult participants from the China Health and Nutrition Survey 2009 was conducted. Logistic regression analyses were used to investigate the relationships between indicators of iron status and the prevalence of unfavorable apolipoprotein profiles. Multivariate linear regression models were constructed to assess the dose-response correlations between serum ferritin and lipid parameters.

**Results:**

After adjustment for confounding factors, in both sexes, the subjects in the top quartile of ferritin had the highest prevalence of an elevated apolipoprotein B (men: odds ratio (OR) 1.97, 95% confidence interval (CI) 1.50–2.62; women: OR 2.13, 95% CI 1.53–2.97) and an elevated apolipoprotein B/apolipoprotein A1 ratio (men: OR 2.00, 95% CI 1.50–2.66; women: OR 1.41, 95% CI 1.04–1.92) when compared with individuals in the lowest quartile. Hemoglobin were also independently associated with unfavorable apolipoprotein B and apolipoprotein B/apolipoprotein A1 ratio both in men and women. However, transferrin (men: OR 0.74, 95% CI 0.56–0.99; women: OR 0.73, 95% CI 0.56–0.95) and soluble transferrin receptor (men: OR 0.75, 95% CI 0.57–0.99; women: OR 0.71, 95% CI 0.55–0.91) were found to be negatively associated with a decreased apolipoprotein A1. Moreover, after controlling for potential confounders, the ferritin concentrations were significantly associated with the levels of lipid ratios including TG/HDL-C, non-HDL-C/HDL-C, TC/HDL-C, apoB/apoA1, and LDL-C/HDL-C ratio in men (β coefficient = 0.147, 0.061, 0.043, 0.038, 0.032, respectively, all *P* values < 0.001) and in women (β coefficient = 0.074, 0.034, 0.025, 0.020, 0.018, respectively, all *P* values < 0.05).

**Conclusions:**

The indicators of iron status are significantly associated with unfavorable apolipoprotein profiles. Serum ferritin concentrations are positively correlated with the levels of lipid ratios. The management on the modifiable iron status and lipid metabolism has a clinical significance. The atherosclerotic lipid profiles of the patients with iron overload deserve special clinical concerns.

## Background

Apolipoproteins, the structural components of lipoproteins, play an essential role in lipid transportation and metabolism [1]. Increasing evidence has shown that elevated levels of serum apolipoprotein B100 (apoB), as well as decreased levels of serum apolipoprotein A1 (apoA1), are both independent risk factors for cardiovascular diseases (CVD) [2–5]. The concentration of circulating apoB provides a measure of all potentially atherogenic lipoproteins, including intermediate-density lipoprotein, very low-density lipoprotein, and low-density lipoprotein (LDL), particularly the small dense LDL particles [6, 7]. Results from recent studies have generally demonstrated the superiority of apoB over a traditional lipid profile (total cholesterol (TC), low-density lipoprotein cholesterol (LDL-C), high-density lipoprotein cholesterol (HDL-C), and non-high-density lipoprotein cholesterol (non-HDL-C)) for predicting cardiovascular risk [8–10]. Moreover, there is growing evidence that the level of apoB is a better monitor of the efficacy of statin therapy, particularly in individuals with normal or low LDL-C levels [11]. As the major constituent of high density lipoprotein particles, apoA1 functions in the process of reverse transportation of cholesterol, as well as anti-inflammatory and antioxidant processes [12]. However, data that compare apoA1 and HDL-C have been inconsistent [13]. Accordingly, the ratio of apoB/apoA1 seems to reflect the status of pro- and anti-atherogenic lipoproteins in a simple manner, but the comparison with conventional lipid ratios is particularly complex. In addition, data of epidemiological studies have indicated that unfavorable apolipoprotein profiles are potentially associated with the metabolic disorders and complications (including insulin resistance (IR) [14], metabolic syndrome (MetS) [15], diabetes [16], polycystic ovary syndrome [17]), and cancers [18].

Iron is a crucial element for human survival, but exposure to excess iron has been found to provoke diseases due to its role in pathological catalytic reactions or oxidative stress. The iron homeostasis means the well-regulation of iron entry, recycling, storage and disposal in line with the adequate amounts for health requirements. Serum ferritin (SF), maintaining the excess iron in a safe and bioavailable form, has been demonstrated to correlate linearly to bodily iron stores [19, 20]. Although serum iron is widely used as an index of functional iron, SF is a more sensitive index of iron overload for residual iron stores [21]. An increasing amount of data have manifested correlations between SF and increased risk of hypertension [22], IR [23], diabetes [24], MetS [25], and CVD [26]. Studies have also debated the relationship between SF and lipid metabolism [27, 28]. Hence, apolipoproteins and lipid ratios are hypothesized to be associated with iron status markers. However, as a note of caution, the synthesis of SF can also be affected by inflammation, so it is still inconclusive whether the associations of SF with lipid parameters were observed due to the iron overload or the influence of inflammation [29]. Additionally, data focused on the association of iron status, which involves SF, transferrin (TRF), soluble transferrin receptor (sTfR), and hemoglobin (Hb), with apolipoproteins and lipid ratios are still rare.

To evaluate the relationships of iron status with apolipoproteins and lipid ratios based on the hypothesis aforementioned, a cross-sectional analysis on the nationwide population-based data is conducted.

## Methods

### Study design

The China Health and Nutrition Survey (CHNS) is a prospective cohort study. A total of ten waves were conducted in 1989, 1991,1993, 1997, 2000, 2004, 2006, 2009, 2011, and 2015. This study used the data of the 2009 wave since blood samples were initially measured. In this wave, a stratified multistage, random cluster process was performed to draw samples from nine provinces, that constituted 47% of China’s population [30]. Details regarding CHNS are available at the World Wide Web site (http://www.cpc.unc.edu/projects/china/home.html) and elsewhere [31]. The survey received ethical approval by the Institutional Review Board of the University of North Carolina at Chapel Hill, the National Institute for Nutrition and Food safety at China Center for Disease Control and Prevention, and the Human and Clinical Research Ethics Committee of the China-Japan Friendship Hospital. All participants signed the written informed consents.

### Study population

A total of 8704 adult participants with biomarkers from the 2009 CHNS were enrolled. The exclusion criteria of the present study included pregnancy (*n* = 62), missing data of anthropometry, physical examination, dietary intake, physical activity (*n* = 441), and absence of blood assessment (*n* = 119). Another 285 individuals with hypersensitive C-reactive protein (hs-CRP) > 10 mg/L, which indicates acute inflammation and an artificial SF level [32, 33], and 257 individuals with extreme HDL-C (> 100 mg/dL) or triglycerides (TG) (> 500 mg/dL) were also excluded. Finally, the remaining 7540 participants were included.

### Laboratory measurements

Prior to the blood sample collection, individuals were required to maintain a normal lifestyle for at least 3 days followed by a 8–12 h fasting. Samples (12 mL) were drawn via venipuncture into three 4 mL tubes. According to strict standards, the 500 uL whole blood from the lavender-stoppered tube was collected for on-site routine blood tests in a local laboratory within 3 h. Hb was determined by a LH750 hematology analyzer (Beckman Coulter, Brea, CA, USA). Another 4 mL blood in red-stoppered tube was centrifuged for 15 min with 3000×g. Serum were preserved at − 80 °C for future analyses [33]. The biochemical and immunological detections were completed with strict quality control in the Beijing central laboratory. Temperature control during the delivery process was critical. Hs-CRP, fasting plasma glucose (FPG) and all of the lipids were detected using Hitachi 7600 automated analyzer (Hitachi Inc., Tokyo, Japan). In detail: hs-CRP was detected by the immunoturbidimetric method (Denka Seiken Ltd., Niigata, Japan); FPG was assessed by the glucose oxidase-phenol and aminophenazone method (Randox Laboratories Ltd., Crumlin, UK); apoB and apoA1 were measured using the immunoturbidimetric method (Randox Laboratories Ltd., Crumlin, UK); TG was measured using the glycerol-3-phosphate oxidase-phenol and aminophenazone method (Kyowa, Aichi, Japan); TC was measured using the cholesterol oxidase-phenol and aminophenazone method (Kyowa, Aichi, Japan); LDL-C and HDL-C were determined by the enzymatic method (Kyowa, Aichi, Japan); the concentration of non-HDL-C was calculated as TC minus HDL-C. SF and fasting insulin (FIN) were detected by radioimmunology using the gamma counter XH-6020 (North Institute of Bio-Tech, Beijing, China). The homeostasis model assessment of insulin resistance (HOMA-IR) was calculated as FIN (uIU/mL) × FPG (mmol/L)/22.5. TRF and sTfR were detected via nephelometry using a Siemens BN ProSpec automated analyzer (Siemens, Erlangen, Germany).

### Assessment of covariates

All of the participants were required to provide information, including age, sex, geographic region, nationality, educational attainment, cigarette smoking, and alcohol consumption frequency, using the structured questionnaire. Weights, heights, waist circumferences, and hip circumferences were measured according to strict standards. Body mass indexes (BMIs) were calculated as weights (kg) divided by heights squared (m^2^) for the overweight/obesity diagnosis. Waist-to-hip ratios (WHRs) for central obesity were calculated as measured waist circumference (cm) divided by measured hip circumference (cm).

The metabolic equivalent (MET)-h/week was used to evaluate the level of physical activities based on the information of domestic, occupational, transportation, and leisure activities collected from staff-administered questionnaires [34]. The MET-h/week was calculated as the sum of MET value of each activity multiplied by time spent during a week [35, 36].

The surveys of dietary intake were conducted by well-trained interviewers via collection of both individual and household dietary intakes during three consecutive days. For a 24 h dietary household food assessment, all of the food inventory, which included new food brought into the home, remaining food and wastage amounts, was weighed using a kitchen scale (ranged from 20 g to 15 kg) and recorded. The household consumption was obtained using the changes of the food inventory during an entire day. The individual intake was estimated by asking individuals to tell about all dietary consumed. These items included the types, amounts, and places of meals. In addition, the amounts of individual intake at home were determined by the household food consumed and the personal proportion consumed. Dietary intakes of total energy (kcal), fat (%), and protein (%) were calculated from the mean weights of the three-day individual food consumption and calorie composition from the Chinese Food Composition Table [37, 38].

### Definitions

According to the definition previously published [39, 40], abnormal gender-specific apolipoprotein levels were defined as the apoB ≥85th percentile value (≥ 1.16 g/L in males, ≥ 1.19 g/L in females), the apoA1 <  15th percentile value (< 0.86 g/L in males, < 0.91 g/L in females), and the apoB/apoA1 ratio ≥ 85th percentile value (≥ 1.15 in males, ≥ 1.08 in females).

### Statistical analysis

All statistical analyses were performed using SPSS statistics 24.0 (SPSS Inc., Chicago, IL, USA). Kolmogorov-Smirnov tests were used to determine the data distribution. Due to the skewed distribution, continuous variables were described as medians (interquartile ranges) and were compared using Mann-Whitney U tests between participants with and without abnormal apolipoprotein profiles. Apolipoprotein profiles of participants across the sex-specific quartiles of iron status were compared using Kruskal-Wallis tests. Bonferroni correction was applied to adjust *P* values for multiple comparisons. The categorical variables were described as proportions and were compared using Chi-square tests. Multivariable logistic regression analyses were conducted to calculate the odds ratios (ORs) and 95% confidence intervals (CIs) for assessment on the associations of the iron status (including SF, TRF, sTfR, and Hb) with abnormal apoB, apoA1, and apoB/apoA1 ratio. For each index of iron status, the lowest sex-specific quartile was defined as a reference for comparison. After the variables were natural logarithm (ln)-transformed to approximate normal distributions, multivariable linear regression analyses were conducted to assess the correlations of SF with lipid measurements and ratios. Three models were fitted. Model 1 was without adjustment. Model 2 was adjusted for age, nationality (Han or others), regional area (rural or urban), education level (< high middle school, ≥ high middle school), smoking status (current smoking or not), frequency of drinking (< 3 times/week, ≥ 3 times/week), physical activity (quartile), energy intake (quartile), fat (%, quartile), protein (%, quartile), BMI (< 18.5 kg/m^2^, 18.5–23.9 kg/m^2^, 24.0–27.9 kg/m^2^, ≥ 28 kg/m^2^) [41], WHR (abnormal as > 0.9 in men, > 0.8 in women, or not) [42], and inflammation status (hs-CRP <  1 mg/L, 1–3 mg/L, and 4–10 mg/L) [43]. Model 3 was adjusted for all of the variables in model 2, plus the HOMA-IR (quartile). The variance inflation factor < 5 was used to exclude the potential multicollinearity for each independent variable in models. A two-tailed *P* <  0.05 was considered statistically significant.

## Results

### Characteristics of individuals by sex and apolipoprotein profile

Due to the remarkable differences in SF, TRF, sTfR, and Hb levels between sexes, following analyses were performed separately by sex. Table 1 shows the gender-specific characteristics of participants grouped by apolipoprotein profiles. Compared with the participants without elevated apoB, in both genders, individuals with elevated apoB were older, more likely to be from urban areas, had less MET-hours/week and higher levels of BMI, WHR, fat (%) and protein (%) intake, SF, Hb, hs-CRP, and HOMA-IR (*P* <  0.05). Higher TRF concentrations and drinking frequency were only displayed in male participants with abnormal apoB, whereas a higher percentage of smoking and lower total energy intake were only displayed in females (*P* <  0.05). Individuals with decreased apoA1 were younger, more likely to be of Han nationality, had higher BMI and hs-CRP levels than those without decreased apoA1in both genders (*P* <  0.05). Higher SF and Hb levels were only displayed in males and lower sTfR levels only in females with decreased apoA1 (*P* <  0.05).

**Table 1:** Characteristics of the study population according to abnormal apolipoprotein profiles

	Apolipoprotein B, g/L			Apolipoprotein A1, g/L			Apolipoprotein B/ Apolipoprotein A1		
**Men (*n*** **= 3481)**	≥ 1.16 (*n* = 549)	< 1.16 (*n* = 2932)	*P*	< 0.86 (*n* = 510)	≥ 0.86 (*n* = 2971)	*P*	≥ 1.15 (*n* = 520)	< 1.15 (*n* = 2961)	*P*
Age, year	53.56 (44.81, 60.96)	50.96 (39.09, 61.66)	< 0.001	46.20 (36.04, 58.87)	52.01 (40.74, 61.87)	< 0.001	51.42 (41.31, 61.67)	51.27 (39.68, 61.51)	0.183
Han nationality, %	88.2	89.3	0.421	95.5	88.1	< 0.001	92.1	88.6	0.018
Rural area, %	62.3	71.6	< 0.001	62.5	71.4	< 0.001	59.8	71.9	< 0.001
Education < high school, %	66.5	72.7	0.003	65.5	72.8	0.001	67.3	72.5	0.016
Smoking, %	54.1	54.6	0.816	51.6	55.1	0.143	56.2	54.3	0.427
Drink frequency ≥ 3/week, %	32.2	24.6	< 0.001	18.8	27.0	< 0.001	25.0	25.9	0.652
Body mass index, kg/m^2^	24.6 (22.5, 26.7)	22.8 (20.6, 25.2)	< 0.001	24.5 (21.8, 26.6)	22.9 (20.7, 25.2)	< 0.001	25.0 (23.0, 27.2)	22.7 (20.6, 25.1)	< 0.001
Waist-to-hip ratio	0.91 (0.87, 0.95)	0.88 (0.84, 0.93)	< 0.001	0.90 (0.85, 0.94)	0.89 (0.84, 0.93)	0.002	0.91 (0.87, 0.95)	0.88 (0.84, 0.93)	< 0.001
MET-hours/week	81.9 (23.8, 171.8)	102.2 (26.3, 210.5)	0.008	90.9 (22.5, 206.1)	99.4 (26.6, 207.0)	0.459	81.3 (22.1, 166.9)	102.5 (26.6, 212.0)	0.001
Energy intake, kcal/day	2234.7 (1780.42720.3)	2281.2 (1880.32716.1)	0.138	2293.5 (1886.52665..4)	2273.3 (1863.82723.3)	0.560	2252.3 (1808.12686.1)	2277.5 (1875.6, 2721.3)	0.155
Fat, %	32.3 (25.2, 39.0)	29.9 (23.1, 37.2)	< 0.001	30.8 (23.2, 38.7)	30.2 (23.4, 37.5)	0.550	31.6 (25.0, 38.6)	30.0 (23.1, 37.3)	0.003
Protein, %	12.5 (10.8, 14.5)	12.0 (10.5, 13.9)	0.001	12.5 (10.8, 14.3)	12.0 (10.5, 14.0)	0.001	12.6 (10.9, 14.5)	12.0 (10.5, 13.9)	< 0.001
HOMA-IR	2.86 (1.87, 4.41)	2.27 (1.52, 3.43)	< 0.001	2.72 (1.79, 4.62)	2.29 (1.54, 3.45)	< 0.001	3.07 (1.96, 5.11)	2.26 (1.52, 3.37)	< 0.001
Hs-CRP, mg/L	2.0 (1.0, 3.0)	1.0 (0.0, 2.0)	< 0.001	1.0 (1.0, 2.0)	1.0 (1.0, 2.0)	0.004	2.0 (1.0, 3.0)	1.0 (0.0, 2.0)	< 0.001
Serum ferritin, μg/L	150.9 (93.3, 267.9)	113.4 (70.8, 190.3)	< 0.001	137.8 (76.3, 258.3)	116.8 (73.1, 193.4)	< 0.001	157.5 (96.5, 323.1)	113.1 (71.0, 187.1)	< 0.001
Transferrin, g/L	2.85 (2.54, 3.23)	2.74 (2.45, 3.07)	< 0.001	2.73 (2.44, 3.10)	2.76 (2.46, 3.10)	0.534	2.85 (2.54, 3.20)	2.74 (2.45, 3.07)	< 0.001
sTfR, mg/L	1.31 (1.08, 1.60)	1.27 (1.05, 1.55)	0.092	1.27 (1.01, 1.53)	1.28 (1.06, 1.56)	0.054	1.30 (1.03, 1.58)	1.27 (1.06, 1.55)	0.991
Hemoglobin, g/L	156.0 (147.0, 165.0)	151.0 (142.0, 161.0)	< 0.001	153.0 (143.0, 163.0)	152.0 (142.0, 162.0)	0.038	155.0 (146.3, 165.0)	152.0 (142.0, 162.0)	< 0.001
**Women (*n*** **= 4059)**	≥ 1.19 (*n* = 610)	< 1.19 (*n* = 3499)	*P*	< 0.91 (*n* = 573)	≥ 0.91 (*n* = 3486)	*P*	≥ 1.08 (*n* = 604)	< 1.08 (*n* = 3455)	*P*
Age, year	58.96 (52.09, 67.15)	48.19 (39.0, 59.32)	< 0.001	47.57 (38.35, 59.68)	51.51 (40.88, 61.28)	< 0.001	57.10 (49.0, 66.27)	49.10 (39.16, 59.84)	< 0.001
Han nationality, %	88.5	88.9	0.805	93.4	88.1	< 0.001	91.2	88.4	0.042
Rural area, %	63.0	70.0	0.001	66.8	69.2	0.249	60.9	70.3	< 0.001
Education < high school, %	84.9	79.5	0.002	80.3	80.3	0.994	84.3	79.6	0.008
Smoking, %	5.1	3.4	0.04	4.5	3.5	0.219	6.5	3.2	< 0.001
Drink frequency ≥ 3/week, %	2.1	1.9	0.683	2.1	1.9	0.745	2.2	1.9	0.654
Body mass index, kg/m^2^	24.4 (22.2, 26.8)	22.8 (20.7, 25.2)	< 0.001	24.2 (21.8, 26.5)	22.9 (20.8, 25.3)	< 0.001	24.6 (22.4, 27.2)	22.8 (20.7, 25.2)	< 0.001
Waist-to-hip ratio	0.88 (0.84, 0.92)	0.85 (0.80, 0.90)	< 0.001	0.86 (0.81, 0.91)	0.86 (0.81, 0.90)	0.159	0.88 (0.84, 0.92)	0.85 (0.81, 0.90)	< 0.001
MET-hours/week	65.6 (38.0, 127.2)	89.2 (47.0, 174.9)	< 0.001	84.8 (47.6, 167.1)	85.0 (44.9, 168.3)	0.519	67.7 (38.5, 129.0)	88.9 (47.0, 174.4)	< 0.001
Energy intake, kcal/day	1861.4 (1498.12240.4)	1928.6 (1573.32293.4)	0.011	1995.8 (1595.7, 2332.0)	1899.0 (1553.22272.9)	0.004	1850.4 (1530.82242.2)	1928.6 (1567.82289.9)	0.155
Fat, %	32.8 (25.3, 39.6)	31.1 (24.0, 38.2)	0.003	31.3 (23.5, 38.0)	31.4 (24.2, 38.7)	0.466	32.0 (24.7, 39.2)	31.2 (24.1, 38.4)	0.131
Protein, %	12.5 (10.7, 14.4)	12.2 (10.5, 14.2)	0.042	12.2 (10.6, 14.2)	12.2 (10.5, 14.2)	0.822	12.4 (10.6, 14.4)	12.2 (10.5, 14.2)	0.251
HOMA-IR	3.04 (2.01, 4.76)	2.25 (1.59, 3.36)	< 0.001	2.53 (1.67, 3.90)	2.33 (1.64, 3.50)	0.083	2.99 (1.94, 4.58)	2.26 (1.60, 3.38)	< 0.001
Hs-CRP, mg/L	2.0 (1.0, 4.0)	1.0 (0.0, 2.0)	< 0.001	1.0 (1.0, 2.0)	1.0 (0.0, 2.0)	0.002	2.0 (1.0, 4.0)	1.0 (0.0, 2.0)	< 0.001
Serum ferritin, μg/L	82.05 (47.05, 140.52)	45.91 (21.78, 83.90)	< 0.001	44.95 (22.14, 88.76)	51.3 (24.1, 91.90)	0.129	72.17 (39.39, 125.29)	47.01 (22.32, 86.71)	< 0.001
Transferrin, g/L	2.93 (2.59, 3.28)	2.87 (2.57, 3.25)	0.169	2.84 (2.57, 3.23)	2.89 (2.57, 3.26)	0.447	2.88 (2.59, 3.24)	2.88 (2.57, 3.26)	0.626
sTfR, mg/L	1.42 (1.19, 1.72)	1.39 (1.14, 1.73)	0.324	1.35 (1.08, 1.73)	1.40 (1.16, 1.73)	0.020	1.38 (1.10, 1.68)	1.40 (1.15, 1.74)	0.082
Hemoglobin, g/L	135.0 (126.8, 143.0)	131.0 (122.0, 140.0)	< 0.001	131.0 (121.0, 140.0)	132.0 (123.0, 140.0)	0.103	135.0 (126.0, 143.0)	131.0 (123.0, 140.0)	< 0.001

Data are presented as medians (interquartile range) or percentages

*MET* metabolic equivalent, *HOMA-IR* homeostasis model assessment of insulin resistance, *Hs-CRP* hypersensitive C-reactive protein, *sTfR* soluble transferrin receptor

**Fig. 1:**
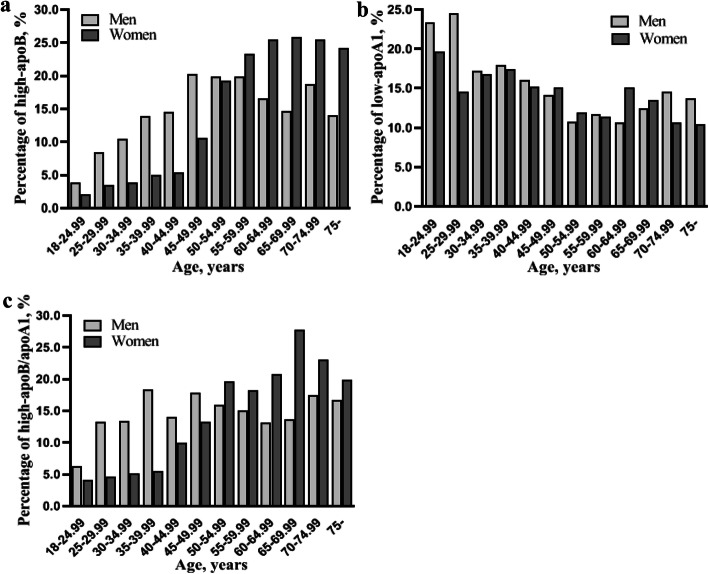
The crude prevalence of an unfavorable apolipoprotein profile according to age and gender. **a** The prevalence of high-apoB increased along with age in both genders. (all *P* values < 0.001). **b** The prevalence of low-apoA1 decreased along with age in both genders. (all *P* values < 0.001). **c** The prevalence of a high-apoB/apoA1 ratio increased along with age in the female samples (*P* value < 0.001), whereas the trend did not present in the male participants (*P* value = 0.091)

Figure 1 shows the prevalence of abnormal apoB, apoA1 and apoB/apoA1 ratio according to age and gender. In both genders, an increased prevalence of unfavorable apoB and a decreased prevalence of unfavorable apoA1 were found along with age (all *P* <  0.001). For the prevalence of low-apoA1 in women, a slight peak occurred during the ages of 60–64.99 years. The prevalence of a high-apoB/apoA1 ratio increased with age in the female samples (*P* <  0.001), whereas the tendency was not significant in the male participants (*P* = 0.091).

### Apolipoprotein profiles of participants across sex-specific quartiles of iron status

In both sexes, participants in the highest quartiles of SF and Hb had the higher levels of apoB and apoB/apoA1 ratios than those in the lowest quartiles (*P* <  0.05), and higher apoA1 level was shown in the highest quartile of sTfR compared with the lowest quartile (*P* <  0.05). More details regarding comparison of apolipoprotein profiles across the sex-specific quartiles of iron status are shown in Table 2.

**Table 2:** Apolipoprotein profiles of participants across sex-specific quartiles of iron status

	Men (***n*** = 3481)					Women (***n*** = 4059)				
		n	ApoB, g/L	ApoA1, g/L	ApoB/ApoA1		n	ApoB, g/L	ApoA1, g/L	ApoB/ApoA1
SF, μg/L										
Q1	51.9 (37.8, 62.8)	872	0.82 (0.68, 1.00)	1.08 (0.93, 1.26)	0.75 (0.59, 0.94)	13.0 (7.4, 18.5)	1015	0.78 (0.65, 0.94)	1.10 (0.96, 1.27)	0.69 (0.56, 0.85)
Q2	95.5 (84.3, 106.8)	869	0.86 (0.71, 1.02)	1.09 (0.94, 1.27)	0.77 (0.62, 0.97)	35.5 (29.0, 43.2)	1015	0.84 (0.69, 1.01)^*^	1.11 (0.97, 1.28)	0.73 (0.58, 0.92)^*^
Q3	147.9 (132.6, 169.5)	870	0.90 (0.74, 1.09)^*#^	1.09 (0.93, 1.28)	0.82 (0.64, 1.03)^*#^	67.5 (58.6, 78.0)	1015	0.93 (0.76, 1.11)^*#^	1.13 (1.00, 1.31)^*^	0.79 (0.63, 0.98)^*#^
Q4	379.9 (253.2, 609.0)	870	0.95 (0.78, 1.13)^*#†^	1.05 (0.88, 1.25)^*#†^	0.92 (0.71, 1.14)^*#†^	140.2 (109.7, 203.3)	1014	0.99 (0.81, 1.20)^*#†^	1.13 (0.98, 1.31)	0.86 (0.69, 1.06)^*#†^
TRF, g/L										
Q1	2.25 (2.10, 2.36)	831	0.83 (0.69, 0.99)	1.06 (0.91, 1.27)	0.76 (0.59, 0.95)	2.35 (2.18, 2.46)	957	0.88 (0.71, 1.05)	1.12 (0.97, 1.30)	0.76 (0.61, 0.97)
Q2	2.60 (2.52, 2.66)	857	0.87 (0.72, 1.04)^*^	1.08 (0.92, 1.28)	0.79 (0.63, 1.01)	2.71 (2.64, 2.78)	1029	0.88 (0.73, 1.06)	1.12 (0.97, 1.29)	0.78 (0.62, 0.95)
Q3	2.91 (2.82, 3.01)	977	0.89 (0.73, 1.07)^*^	1.08 (0.93, 1.25)	0.83 (0.64, 1.04)^*^	3.05 (2.95, 3.14)	1036	0.88 (0.72, 1.09)	1.12 (0.98, 1.30)	0.76 (0.61, 0.97)
Q4	3.35 (3.22, 3.60)	816	0.93 (0.77, 1.13)^*#†^	1.08 (0.92, 1.28)	0.86 (0.68, 1.10)^*#†^	3.56 (3.38, 3.85)	1037	0.87 (0.72, 1.08)	1.12 (0.99, 1.30)	0.76 (0.60, 0.95)
sTfR, mg/L										
Q1	0.90 (0.80, 0.98)	835	0.86 (0.71, 1.05)	1.04 (0.90, 1.21)	0.82 (0.64, 1.05)	0.97 (0.86, 1.06)	989	0.87 (0.71, 1.05)	1.08 (0.95, 1.25)	0.78 (0.62, 0.98)
Q2	1.15 (1.10, 1.21)	839	0.86 (0.72, 1.03)	1.10 (0.94, 1.27)^*^	0.78 (0.61, 0.97)^*^	1.19 (1.26, 1.31)	926	0.87 (0.72, 1.05)	1.12 (0.98, 1.30)^*^	0.75 (0.60, 0.93)
Q3	1.39 (1.32, 1.46)	905	0.88 (0.73, 1.07)	1.08 (0.93, 1.28)^*^	0.80 (0.64, 1.03)	1.52 (1.44, 1.62)	1156	0.91 (0.73, 1.10)^*^	1.14 (0.99, 1.31)^*^	0.78 (0.61, 0.98)
Q4	1.82 (1.67, 2.12)	902	0.92 (0.74, 1.09)^*#^	1.08 (0.92, 1.29)^*^	0.83 (0.64, 1.05)^#^	2.09 (1.88, 2.51)	988	0.87 (0.72, 1.06)	1.14 (0.98, 1.33)^*^	0.74 (0.60, 0.93)
Hb, g/L										
Q1	134.0 (126.0, 139.0)	871	0.82 (0.68, 0.99)	1.08 (0.94, 1.27)	0.75 (0.58, 0.94)	116.0 (109.0, 120.0)	1056	0.83 (0.68, 1.00)	1.13 (0.96, 1.30)	0.72 (0.58, 0.90)
Q2	148.0 (145.0, 150.3)	906	0.87 (0.70, 1.04)^*^	1.08 (0.92, 1.28)	0.79 (0.61, 1.01)^*^	128.0 (126.0, 130.0)	1089	0.86 (0.71, 1.03)^*^	1.11 (0.98, 1.30)	0.76 (0.60, 0.93)^*^
Q3	157.0 (155.0, 160.0)	869	0.92 (0.76, 1.07)^*#^	1.07 (0.92, 1.25)	0.84 (0.66, 1.07)^*#^	136.0 (134.0, 138.0)	929	0.90 (0.73, 1.10)^*#^	1.12 (0.99, 1.32)	0.78 (0.62, 0.99)^*#^
Q4	165.0 (169.0, 177.0)	835	0.92 (0.76, 1.13)^*#^	1.05 (0.91, 1.26)	0.86 (0.68, 1.08)^*#^	147.0 (143.0, 155.0)	985	0.93 (0.76, 1.13)^*#^	1.12 (0.97, 1.28)	0.81 (0.64, 1.01)^*^

Data are presented as medians (interquartile range)

*ApoB* apolipoprotein B, *ApoA1* apolipoprotein A1, *SF* serum ferritin, *TRF* transferrin, *sTfR* soluble transferrin receptor, *Hb* hemoglobin, *Q* quartile

^*^P < 0.05 compared with the reference Q1; ^#^*P* < 0.05 compared with the reference Q2; ^†^*P* < 0.05 compared with the reference Q3

### Associations of iron status with the prevalence of unfavorable apolipoprotein profiles

To study the sex-specific correlations of SF, TRF, sTfR, and Hb with abnormal apolipoprotein profiles, the comparative ORs and 95% CIs of the highest quartile are shown in Table 3. In both genders, after controlling for age, nationality, regional area, education, smoking, alcohol frequency, physical activity, diet, BMI, WHR, and hs-CRP (model 2), SF values were positively correlated with elevated apoB and apoB/apoA1 ratios (Table 3). When further adjusted for HOMA-IR (model 3), the relationship still maintained significance (men: OR for apoB 1.97, 95% CI 1.50–2.62; OR for apoB/apoA1 2.00, 95% CI 1.50–2.66; women: OR for apoB 2.13, 95% CI 1.53–2.97; OR for apoB/apoA1 1.41, 95% CI 1.04–1.92). Hb values were significantly associated with unfavorable apoB and apoB/apoA1 ratios in both sexes (men: OR for apoB 2.04, 95% CI 1.51–2.74; OR for apoB/apoA1 1.39, 95% CI 1.03–1.87. women: OR for apoB 1.71, 95% CI 1.31–2.25; OR for apoB/apoA1 1.55, 95% CI 1.18–2.02), whereas sTfR values were associated with neither apoB nor apoB/apoA1 ratios. The positive correlation between TRF and elevated apoB was observed in both sexes (men: OR 2.00, 95% CI 1.50–2.66; women: OR 1.55, 95% CI 1.18–2.04). However, no significant association of TRF with elevated apoB/apoA1 ratio was found in men (men: OR 1.62, 95% CI 1.21–2.16; women: OR 1.21, 95% CI 0.92–1.59). In addition, the negative associations of TRF and sTfR with decreased apoA1 were significant in both sexes (TRF, men: OR 0.74, 95% CI 0.56–0.99; women: OR 0.73, 95% CI 0.56–0.95; sTfR, men: OR 0.75, 95% CI 0.57–0.99; women: OR 0.71, 95% CI 0.55–0.91). The association between Hb and decreased apoA1 was significant only in women (OR 0.70, 95% CI 0.54–0.91).

**Table 3:** Odds ratios of abnormal apolipoprotein profiles in the extreme sex-specific quartiles of iron status

	ApoB			ApoA1			ApoB/ApoA1		
	OR^a^ (95% CI)	OR^b^ (95% CI)	OR^c^ (95% CI)	OR^a^ (95% CI)	OR^b^ (95% CI)	OR^c^ (95% CI)	OR^a^ (95% CI)	OR^b^ (95% CI)	OR^c^ (95% CI)
**Men**									
SF	2.54 (1.94, 3.33)	2.04 (1.54, 2.71)	1.97 (1.50, 2.62)	1.52 (1.18, 1.95)	1.22 (0.93, 1.59)	1.19 (0.91, 1.55)	2.78 (2.12, 3.65)	2.08 (1.56, 2.78)	2.00 (1.50, 2.66)
TRF	2.11 (1.61, 2.75)	2.10 (1.58, 2.78)	2.00 (1.50, 2.66)	0.99 (0.76, 1.30)	0.77 (0.58, 1.03)	0.74 (0.56, 0.99)	1.93 (1.47, 2.52)	1.73 (1.29, 2.30)	1.62 (1.21, 2.16)
sTfR	1.27 (0.98, 1.64)	1.32 (1.01, 1.72)	1.28 (0.97, 1.67)	0.74 (0.57, 0.96)	0.77 (0.59, 1.00)	0.75 (0.57, 0.99)	0.93 (0.72, 1.20)	0.96 (0.74, 1.26)	0.92 (0.70, 1.21)
Hb	2.18 (1.66, 2.87)	2.14 (1.59, 2.87)	2.04 (1.51, 2.74)	1.18 (0.90, 1.55)	0.84 (0.62, 1.12)	0.81 (0.61, 1.09)	1.78 (1.36, 2.35)	1.47 (1.09, 1.98)	1.39 (1.03, 1.87)
**Women**									
SF	5.59 (4.17, 7.50)	2.27 (1.64, 3.16)	2.13 (1.53, 2.97)	0.86 (0.67, 1.09)	0.97 (0.72, 1.30)	0.96 (0.72, 1.29)	3.21 (2.46, 4.19)	1.49 (1.10, 2.03)	1.41 (1.04, 1.92)
TRF	1.10 (0.86, 1.41)	1.72 (1.31, 2.25)	1.55 (1.18, 2.04)	0.93 (0.72, 1.19)	0.75 (0.57, 0.97)	0.73 (0.56, 0.95)	0.99 (0.77, 1.27)	1.31 (1.00, 1.71)	1.21 (0.92, 1.59)
sTfR	1.12 (0.87, 1.44)	1.28 (0.98, 1.68)	1.26 (0.96, 1.66)	0.72 (0.56, 0.91)	0.72 (0.56, 0.92)	0.71 (0.55, 0.91)	0.79 (0.61, 1.01)	0.88 (0.68, 1.15)	0.87 (0.67, 1.13)
Hb	1.95 (1.52, 2.50)	1.86 (1.42, 2.43)	1.71 (1.31, 2.25)	0.90 (0.71, 1.15)	0.73 (0.56, 0.94)	0.70 (0.54, 0.91)	1.88 (1.47, 2.41)	1.64 (1.26, 2.14)	1.55 (1.18, 2.02)

*ApoB* apolipoprotein B, *ApoA1* apolipoprotein A1, *OR* odds ratio, *CI* confidence interval, *SF* serum ferritin, *TRF* transferrin, *sTfR* soluble transferrin receptor, *Hb* hemoglobin

^a^Original model without any adjustments. ^b^Adjusted for age, nationality, regional area, education level, smoking status, alcohol frequency, physical activity, energy intake, fat% intake, protein% intake, body mass index, waist-to-hip ratio and hypersensitive C-reactive protein. ^c^Adjusted for all the variables in model 2 plus the homeostasis model assessment of insulin resistance. Likelihood ratio Chi-square testing was implemented for models comparison in multivariable logistic regression (*P* < 0.001)

### Correlations of SF levels with lipid measurements and lipid ratios

The results of the multivariable linear regression analyses that assessed the correlations between SF levels and lipid parameters are shown in Table 4. After controlling for potential confounders, in both genders, the levels of apoB, TG, TC, LDL-C, and non-HDL-C were positively associated with the SF concentrations (all *P* values < 0.05). Data from the adjusted models showed, for male participants, the SF concentrations were positively correlated with the levels of TG/HDL-C, non-HDL-C/HDL-C, TC/HDL-C, apoB/apoA1, and LDL-C/HDL-C ratios (β coefficient = 0.147, 0.061, 0.043, 0.038, 0.032, respectively, all *P* values < 0.001). Similarly, the positive associations of SF concentrations with the lipid ratios aformentioned were also shown in females (β coefficient = 0.074, 0.034, 0.025, 0.020, 0.018, respectively, all *P* values < 0.05). However, associations of SF concentration with apoA1 level in both genders and with HDL-C level in women were not significant. More details regarding the dose-response relationship are presented in Table 4.

**Table 4:** β coefficients of lipids variation by sex-specific ln-transformed serum ferritin

	Men			Women		
	β^a^	β^b^	β^c^	β^a^	β^b^	β^c^
**Lipid measures**						
Ln apoB	0.053^†^	0.036^†^	0.034^†^	0.077^†^	0.023^†^	0.021^†^
Ln apoA1	−0.015^*^	−0.005	−0.004	0.008^*^	0.001	0.001
Ln TG	0.185^†^	0.139^†^	0.128^†^	0.146^†^	0.076^†^	0.069^†^
Ln TC	0.032^†^	0.025^†^	0.024^†^	0.052^†^	0.020^†^	0.019^†^
Ln LDL-C	0.023^†^	0.013^†^	0.013^*^	0.060^†^	0.014^†^	0.013^*^
Ln HDL-C	−0.040^†^	−0.021^†^	−0.019^†^	−0.013^†^	− 0.007^*^	−0.006
Ln non-HDL-C	0.062^†^	0.044^†^	0.042^†^	0.080^†^	0.031^†^	0.029^†^
**Lipid ratios**						
Ln apoB/apoA1	0.069^†^	0.041^†^	0.038^†^	0.069^†^	0.023^†^	0.020^†^
Ln TG/HDL-C	0.224^†^	0.160^†^	0.147^†^	0.159^†^	0.083^†^	0.074^†^
Ln TC/HDL-C	0.072^†^	0.047^†^	0.043^†^	0.065^†^	0.027^†^	0.025^†^
Ln LDL-C/HDL-C	0.063^†^	0.035^†^	0.032^†^	0.073^†^	0.021^†^	0.018^*^
Ln non-HDL-C/HDL-C	0.102^†^	0.066^†^	0.061^†^	0.093^†^	0.038^†^	0.034^†^

*Ln* natural logarithm, *apoB* apolipoprotein B, *apoA1* apolipoprotein A1, *TG* triglycerides, *TC* total cholesterol, *LDL-C* low-density lipoprotein cholesterol, *HDL-C* high-density lipoprotein cholesterol, *non-HDL-C* non-high-density lipoprotein cholesterol

^†^*P* < 0.001, ^*^P < 0.05

^a^Original model without any adjustments. ^b^Adjusted for age, nationality, regional area, education level, smoking status, alcohol frequency, physical activity, energy intake, fat% intake, protein% intake, body mass index waist-to-hip ratio and hypersensitive C-reactive protein. ^c^Adjusted for all the variables in model 2 plus the homeostasis model assessment of insulin resistance. F-test was implemented implemented for models comparison in multivariable linear regression (*P* < 0.001)

## Discussion

This study found the following primary results. In both sexes, SF and Hb levels were positively related to the prevalence of elevated apoB and apoB/apoA1, while TRF and sTfR levels were negatively related to decreased apoA1. Those associations were independent of the factors frequently associated with unfavorable apolipoprotein profiles, such as age, nationality, regional area, education, smoking, alcohol frequency, physical activity, diet, BMI, WHR, inflammation status, and HOMA-IR. Furthermore, this study found dose-response relationships of SF concentrations with the levels of a range of lipid parameters including traditional lipid measurements and nontraditional lipid ratios in both sexes.

Previous studies debated on the associations between high SF and dyslipidemic parameters among participants of different ages, sexes, body sizes and ethnic distributions. Kim et al. found that in Korean adolescents, SF was positively correlated with TC, TG, and LDL-C levels only in boys, but negatively correlated with HDL-C in both boys and girls [44]. In a Finnish study of adults, SF was shown to be correlated with markers of cardiometabolic risk (elevated TG, apoB, and reduced HDL-C) [45]. In 2004, Jehn et al. showed that SF was positively correlated with elevated TG in 6044 American adults [46]. Recently, Li et al. showed SF values were significantly associated with TC, LDL-C, TG, and HDL-C independent of diabetes or IR in a Chinese population in both genders [47]. However, Yoo et al. showed that significant associations of high SF with TG and HDL-C in young non-obese adults only existed in women [48]. In fact, the limited data focused on the relationships between SF and lipids except TG and HDL-C. This study extended the previous results by exploring the relationships of SF levels with apolipoprotein profiles and lipid ratios. In addition, the associations of apolipoprotein profiles with TRF, sTfR, and Hb were also investigated. Present data from the adjusted models were stable and independent of age, nationality, area, smoking, drinking, physical activity, diet, obesity, inflammation status, and IR. It was established that elevated apoB and apoB/apoA1 and decreased apoA1 were independently correlated with CVD risk [3, 49]. Hence, the abnormal apolipoprotein profiles associated with unfavorable iron status may predispose individuals with iron overload to the high CVD risk.

Iron exists in circulation mostly in complex with transferrin, ferritin, and heme [50]. As the major carrier and storage proteins, in individuals without iron deficiency, the higher SF, TRF, and Hb levels can reflect a potential capacity of bodily iron within a normal high or even excess degree. Several mechanisms that mediate the link between iron status and lipid metabolism have been proposed. The most frequently proposed mechanism is related to IR. Iron overload has been proven to disturb insulin signaling and cause hyperinsulinemia [51]. An experimental study showed that iron overload in C57b1/6 mice could up-regulate resistin and hepcidin expressions [52], which might in turn induce production of suppressor of cytokine signaling-3, an inhibitor of insulin signaling [53, 54]. Hyperinsulinemia and IR can contribute to dyslipidemia including unfavorable TG, TC, HDL-C and LDL-C. Additionally, hepatic IR is associated with overexpression of protein-tyrosine phosphatase 1B, which in turn contribute to the increased synthesis and secretion of apoB [55]. On one hand, in this study, adjusting for HOMA-IR attenuated the strength of associations between the iron status and abnormal apolipoprotein profiles, as well as the dose-response correlations of SF with the levels of lipid parameters. This implied that IR may play a role in these relationships. On the other hand, the associations remained significant after controlling for HOMA-IR, which also suggested that the relationships could not be entirely explained by IR. Another possible underlying mechanism might be due to oxidative stress. As the first-line prooxidant, the ability of free iron to generate reactive radicals might be involved in dyslipidemia. Previous data have suggested that increased oxidative stress promoted by iron overload could decrease fatty acid oxidation and increase lipid transportation. The results of in vivo rat models have demonstrated that iron dextran could downregulate the expression of peroxisome proliferator activated receptor α and its target genes (Nrf1, cpt-1α), thus decreasing fatty acid oxidation and increasing TG levels [27, 56]. The microsomal triglyceride transfer protein (MTP), which accelerates the apoB production, can be up-regulated in oxidative stress [57]. It has been reported that the expression of MTP also could be up-regulated in iron overloaded rats, resulting in elevated apoB, TC, and TG levels [27]. However, more details of the underlying mechanisms remain to be clarified in humans due to species differences.

It is worth noting that, in this study, SF was not significantly associated with low-apoA1. However, sTfR was negatively associated with decreased apoA1 in both genders, and TRF was negatively associated with decreased apoA1 after adjustment for confounding fators. The sTfR, as a truncated form of transferrin receptor in serum, take part in the process of cellular iron uptake together with TRF. Since the participants were enrolled for a nationwide health and nutrition survey, most samples in this study were healthy, up-regulation of sTfR might reflect cell iron demands rather than iron deficiency throughout the body. Increasing sTfR is potentially associated with the decreased intracellular labile iron pool. Likewise, TRF in plasma, exerts the same type of protecting effect as ferritin in cell, is negatively related to the non-transferrin bound iron. Both the labile iron pool and the unbound iron can promote the production of reactive oxygen species, and in turn induce the lipids oxidation [50]. It is speculated that moderately increased TRF and sTfR may act as an antioxidant reaction to correlated with HDL-C-apoA1 metabolism. The mechanisms behind the links of TRF and sTfR with apoA1 are largely unknown. Both experimental and epidemiological analyses should be performed to furtherly explore these complex associations.

Regarding gender differences, the previous data have been inconsistent. Han et al. demonstrated that subjects with higher SF were significantly related with an elevated risk of unfavorable TG and HDL-C among males. However, higher SF was only associated with elevated TG among females [58]. Kimm et al. also showed a difference in the association of SF with dyslipidemia in 1879 Korean adolescents, while TG, TC, HDL-C, and LDL-C were all associated with SF levels in boys; low HDL-C was the only observed dyslipidemia significantly associated with SF concentration in girls [59]. This study identified gender differences in the prevalence of elevated apoB/apoA1, which increased along with age. Even though a decreased prevalence of low-apoA1 was found along with age in both genders, a slight peak was observed in ages 60–64.99 years in women. Similarly, the AMORIS study of 175,553 Swedish participants reported similar results that apoA1varied by age decade and sex [3]. What’s more, the positive association of TRF level with elevated apoB/apoA1 was only displayed in male participants, and the negative association of Hb level with decreased apoA1 was only displayed in females. Although the reasons for gender differences cannot be clearly elucidated, it is hypothesized that the sex hormones, lifestyle factors, and menopausal status in females may play a part.

### Study strengths and limitations

This study has several strengths, including a large-scale and well-established population-based design and an adjustment for main lifestyle factors. Nevertheless, several limitations also should be considered. First, the cross-sectional study used is not appropriate to draw a causal relationship between the iron status and lipid metabolism. Second, the existence of other confounding factors, such as medical history (lipid lowering therapy and iron supplementation) and menopausal status, cannot be ruled out. A calculated HOMA-IR, but not the IR index of hyperinsulinemic euglycemic clamp used in this study is another limitation.

## Conclusions

In conclusion, SF, TRF, sTfR and Hb, as the markers of iron status, are significantly associated with the prevalence of abnormal apolipoprotein profiles. SF concentration is positively correlated with the levels of lipid ratios. The lipid profiles of the patients with iron overload deserve special clinical concerns. Management targeting the iron excess and associated atherosclerotic dyslipidemia might serve to protect the patients from the cardiometabolic outcomes. Since the role iron status playing, as well as the underlying mechanism behind its relationship with lipid metabolism are still unclear, more prospective and experimental studies are required for further exploration.

## Data Availability

The data and material of this study are available from the author (B.Z.) on reasonable request.

## Abbreviations

apoA1: Apolipoprotein A1
apoB: Apolipoprotein B100
BMI: Body mass index
CHNS: China Health and Nutrition Survey
CI: Confidence interval
CVD: Cardiovascular diseases
FIN: Fasting insulin
FPG: Fasting plasma glucose
Hb: Hemoglobin
HDL-C: High density lipoprotein cholesterol
HOMA-IR: Homeostasis model assessment of insulin resistance
hs-CRP: Hypersensitive C-reactive protein
IR: Insulin resistance
LDL: Low-density lipoprotein
LDL-C: Low density lipoprotein cholesterol
ln: Natural logarithm
MET: Metabolic equivalent
MetS: Metabolic syndrome
MTP: Microsomal triglyceride transfer protein
non-HDL-C: Non-high-density lipoprotein cholesterol
OR: Odds ratio
SF: Serum ferritin
Q: Quartile
sTfR: Soluble transferrin receptor
TC: Total cholesterol
TG: Triglycerides
TRF: Transferrin
WHR: Waist-to-hip ratio
